# Supplementary material for: CC-Chemokine Receptor-2 Expression in Osteoblasts Contributes to Cartilage and Bone Damage during Post-Traumatic Osteoarthritis
Source: Biomolecules. 2023 May 26;13(6):891. doi: 10.3390/biom13060891 (PMC10296290; doi:10.3390/biom13060891)
Supplement: Supplementary file 1 [file biomolecules-13-00891-s001.zip › biomolecules-2316240-supplementary.pdf]

SUPPLEMENTAL FIGURES

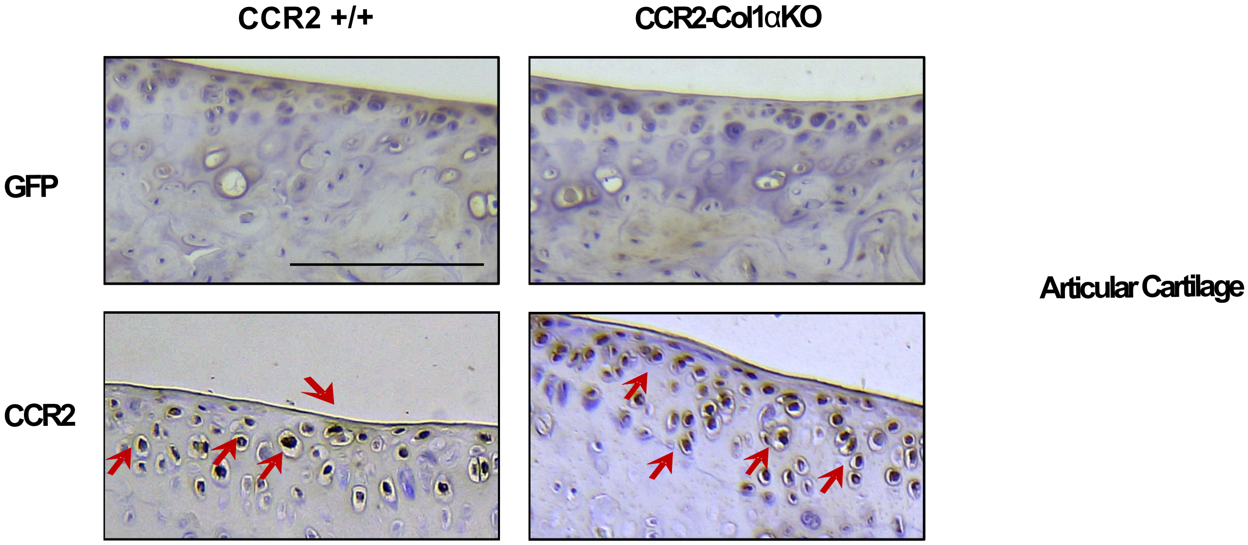


Supplemental Figure S1

**Supplemental Figure S1. Protein levels of CCR2 and GFP in the articular cartilage (AC) of *CCR2-Col1αKO* and *CCR2*+/+ mice following Tamoxifen injection.** Paraffin embedded knee joint sections are immunostained for GFP and CCR2, two weeks after the first Tam injection. Positive staining is detected as brown precipitate (red arrows). GFP staining is not detected in any of the genotypes. Conversely, CCR2 staining is detected in the AC cells of both CCR2+/+ and *CCR2-Col1αKO* mice. Images are representative of 6 different mice for each of the experimental groups described, ranging between 14 and 18 weeks of age. Scale bars are 100 μm.

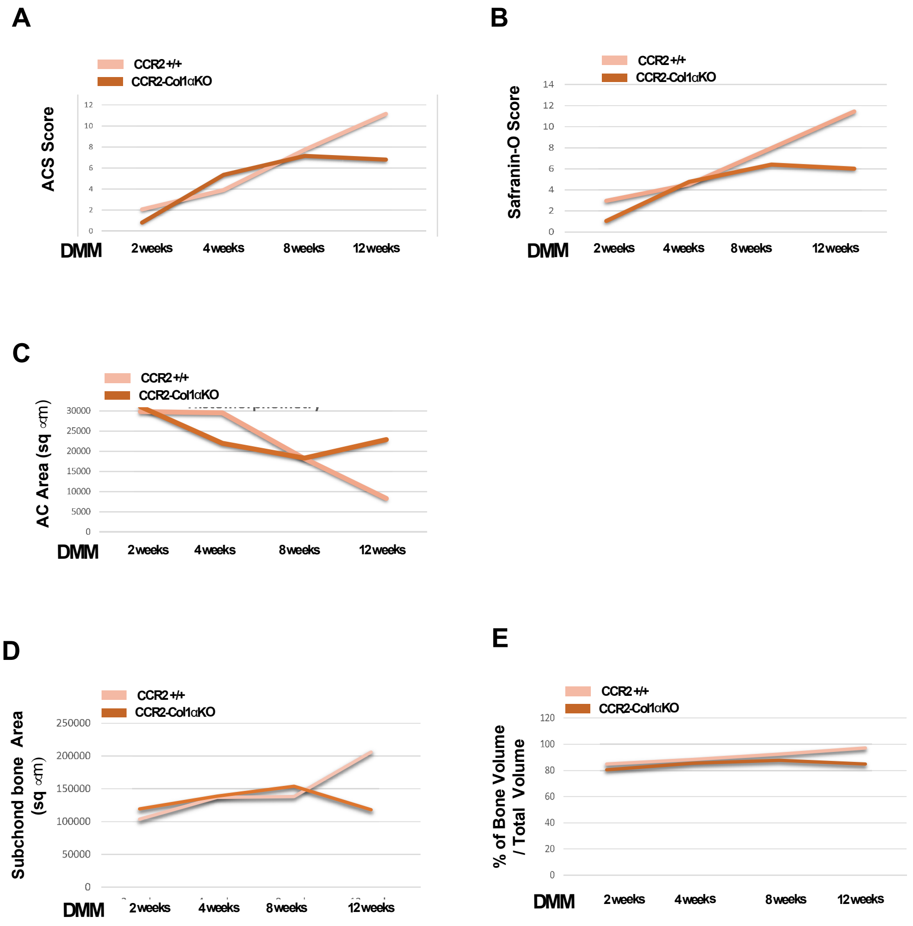


**Supplemental Figure S2. PTOA assessment in mouse knee joints of *CCR2-Col1αKO* and *CCR2+/+* within each genotype, following early Ccr2 inactivation.** ACS, Saf-O score, Histomorphometric analysis of cartilage quantification, Subchondral bone thickness and percentabe of bone volume / Total Volume (BV/TV) were **assessed** at the time point indicated following early Ccr2 inactivation (before DMM). *N*=8 mice for each experimental point. Within each genotype (***CCR2+/+*** **,** light orange; ***CCR2-Col1αKO,*** dark orange), group analyses were performed with ordinary one-way ANOVA, followed by Tukey's post-hoc test for multiple comparisons, available in the Supplemental Table S1.

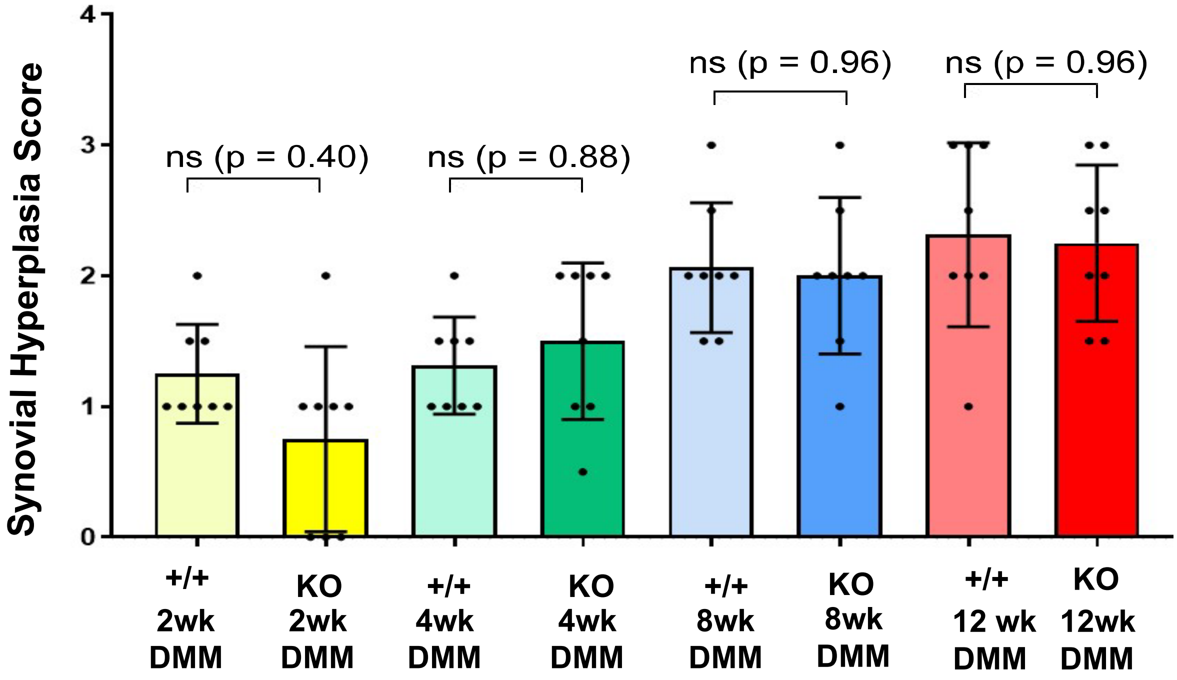


Supplemental Figure S3

**Supplemental Figure S3. Synovial hyperplasia assessment in mouse knee joints of *CCR2-Col1αKO* and *CCR2+/+*, following early CCR2 inactivation.** Synovial hyperplasia scores (scale 0-3) of *CCR2-Col1αKO* and *CCR2+/+* mouse knees at the time point indicated. *N*=8 mice for each experimental point. The graphs represents the mean ± standard deviation; indicated *P*-values were determined by Wilcoxon rank sum tests at each time point, following adjustment for multiple comparisons.

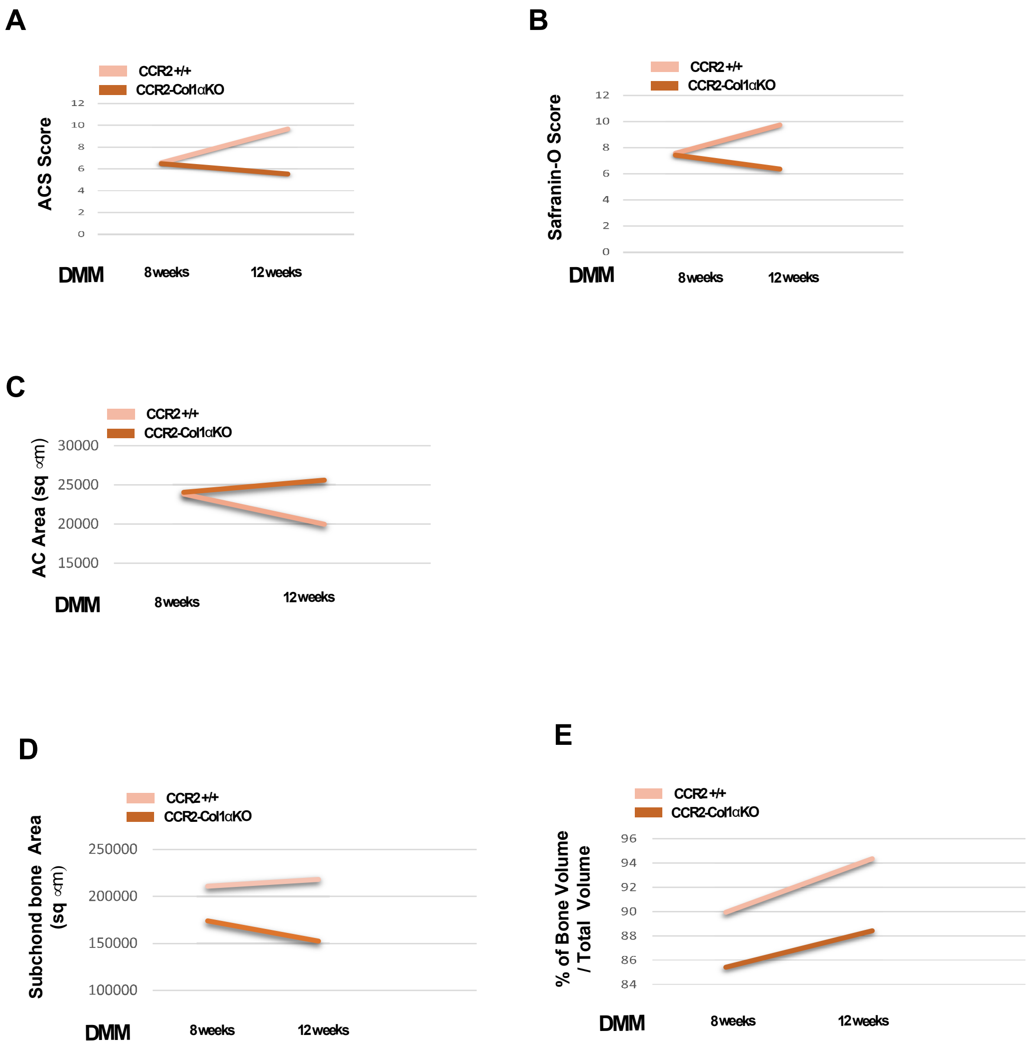


Supplemental Figure S4

**Supplemental Figure S4. PTOA assessment in mouse knee joints of *CCR2-Col1αKO* and *CCR2+/+* within each genotype, following late Ccr2 inactivation.** ACS, Saf-O score, Histomorphometric analysis of cartilage quantification, Subchondral bone thickness and percentabe of bone volume / Total Volume (BV/TV) were **assessed** at the time point indicated following late Ccr2 inactivation (at 4 weeks after DMM). *N*=8 mice for each experimental point. Within each genotype (***CCR2+/+*** **,** light orange; ***CCR2-Col1αKO*,** dark orange), group analyses were performed with Welch's t-test, available in the Supplemental Table S3.

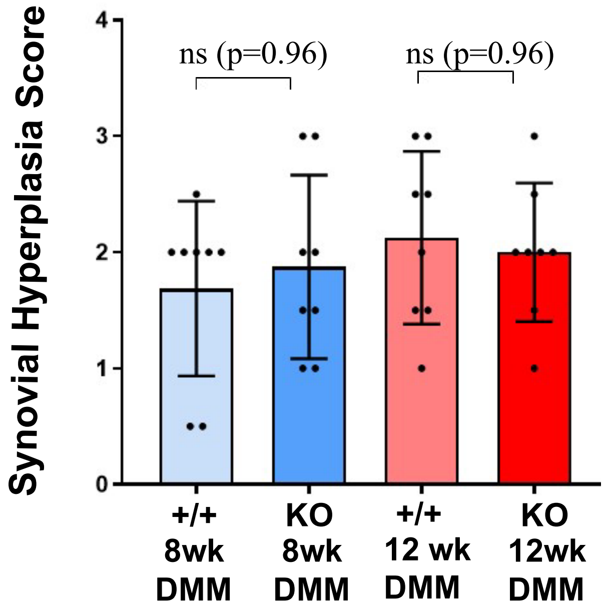


**Supplemental Figure S5**

**Supplemental Figure S5. Synovial hyperplasia assessment in mouse knee joints of *CCR2-Col1αKO* and *CCR2+/+*, following late CCR2 inactivation.** Synovial hyperplasia scores (scale 0-3) of *CCR2-Col1αKO* and *CCR2+/+* mouse knees at the time point indicated. *N*=8 mice for each experimental point. The graphs represents the mean ± standard deviation; indicated p-values were determined by Wilcoxon rank sum tests at each time point, following adjustment for multiple comparisons.

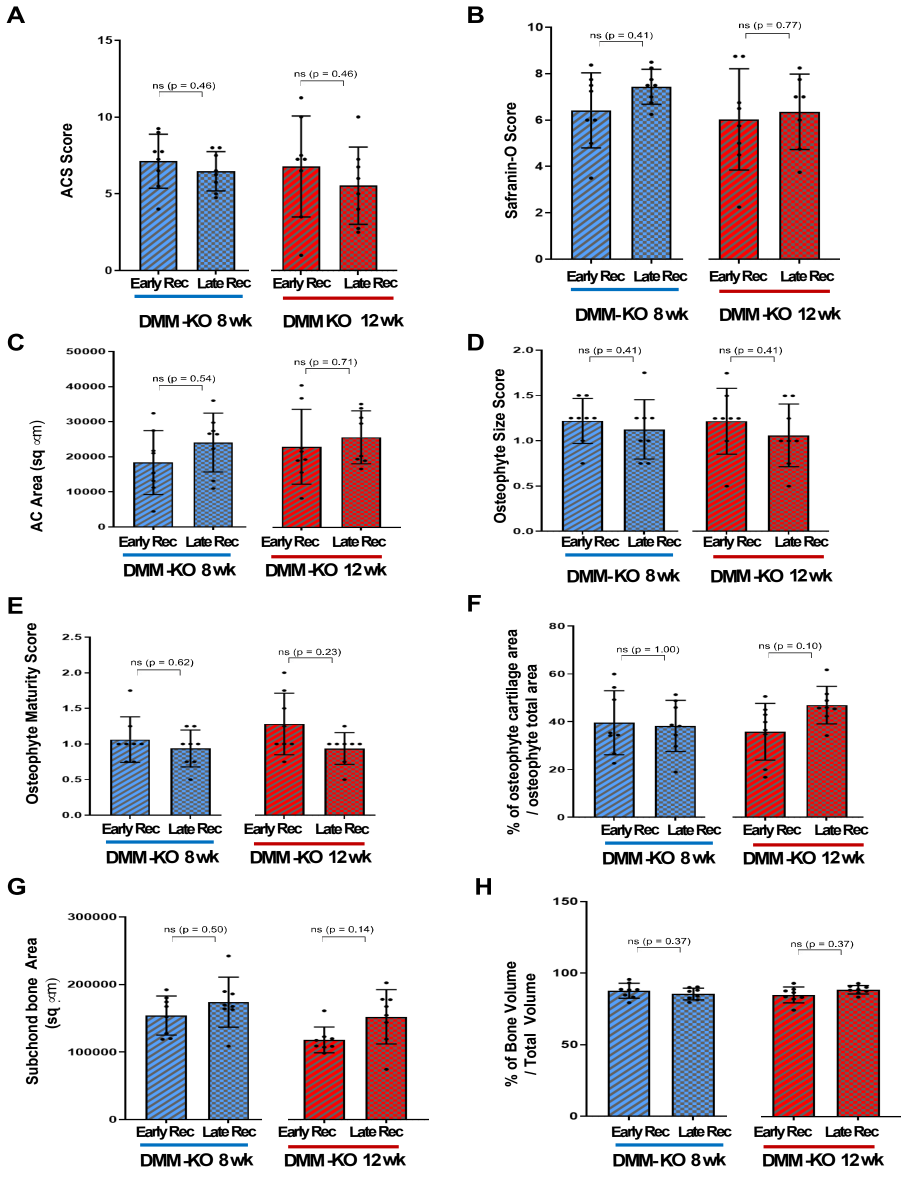


**Supplemental Figure S6. PTOA assessment in mouse knee joints of *CCR2-Col1αKO* comparing early vs late Ccr2 ablation.** ACS, Saf-O score, Histomorphometric analysis of cartilage quantification, Osteophyte Size, Osteophyte maturity, Oateophyte cartilage quantification, Subchondral bone thickness and percentabe of bone volume / Total Volume (BV/TV) were **compared** at 8 and 12 weeks after DMM. *N*=8 mice for each experimental point. The graphs represents the mean ± standard deviation; indicated p-values were determined by Wilcoxon rank sum tests at each time point, following adjustment for multiple comparisons, available in the Supplemental Table S7.

## SUPPLEMENTAL TABLES

| OA Parameter Early Recomb. | Groups | Mean Diff. | 95.00% CI of diff. | Adjusted *P*-value |
|---|---|---|---|---|
| ***ACS*** | DMM 2w CCR2 +/+ vs. DMM 4w CCR2 +/+ | -1.844 | -3.100 to -0.5877 | **0.0022** |
| | DMM 2w CCR2 +/+ vs. DMM 8w CCR2 +/+ | -5.688 | -6.944 to -4.431 | **<0.0001** |
| | DMM 2w CCR2 +/+ vs. DMM 12w CCR2 +/+ | -9.094 | -10.35 to -7.838 | **<0.0001** |
| | DMM 4w CCR2 +/+ vs. DMM 8w CCR2 +/+ | -3.844 | -5.100 to -2.588 | **<0.0001** |
| | DMM 4w CCR2 +/+ vs. DMM 12w CCR2 +/+ | -7.25 | -8.506 to -5.994 | **<0.0001** |
| | DMM 8w CCR2 +/+ vs. DMM 12w CCR2 +/+ | -3.406 | -4.662 to -2.150 | **<0.0001** |
| ***Saf-O*** | DMM 2w CCR2 +/+ vs. DMM 4w CCR2 +/+ | -1.563 | -2.960 to -0.1650 | **0.024** |
| | DMM 2w CCR2 +/+ vs. DMM 8w CCR2 +/+ | -5 | -6.398 to -3.602 | **<0.0001** |
| | DMM 2w CCR2 +/+ vs. DMM 12w CCR2 +/+ | -8.5 | -9.898 to -7.102 | **<0.0001** |
| | DMM 4w CCR2 +/+ vs. DMM 8w CCR2 +/+ | -3.438 | -4.835 to -2.040 | **<0.0001** |
| | DMM 4w CCR2 +/+ vs. DMM 12w CCR2 +/+ | -6.938 | -8.335 to -5.540 | **<0.0001** |
| | DMM 8w CCR2 +/+ vs. DMM 12w CCR2 +/+ | -3.5 | -4.898 to -2.102 | **<0.0001** |
| ***Cartilage Histomorph.*** | DMM 2w CCR2 +/+ vs. DMM 4w CCR2 +/+ | 265.8 | -7257 to 7789 | 0.9997 |
| | DMM 2w CCR2 +/+ vs. DMM 8w CCR2 +/+ | 11657 | 4134 to 19180 | **0.0012** |
| | DMM 2w CCR2 +/+ vs. DMM 12w CCR2 +/+ | 21449 | 13926 to 28972 | **<0.0001** |
| | DMM 4w CCR2 +/+ vs. DMM 8w CCR2 +/+ | 11391 | 3868 to 18914 | **0.0016** |
| | DMM 4w CCR2 +/+ vs. DMM 12w CCR2 +/+ | 21183 | 13660 to 28706 | **<0.0001** |
| | DMM 8w CCR2 +/+ vs. DMM 12w CCR2 +/+ | 9792 | 2269 to 17315 | **0.0071** |
| ***Subchondral Bone Thickness*** | DMM 2w CCR2 +/+ vs. DMM 4w CCR2 +/+ | -33145 | -75391 to 9101 | 0.1647 |
| | DMM 2w CCR2 +/+ vs. DMM 8w CCR2 +/+ | -34614 | -76860 to 7633 | 0.138 |
| | DMM 2w CCR2 +/+ vs. DMM 12w CCR2 +/+ | -102378 | -144624 to -60132 | **<0.0001** |
| | DMM 4w CCR2 +/+ vs. DMM 8w CCR2 +/+ | -1469 | -43715 to 40778 | 0.9997 |
| | DMM 4w CCR2 +/+ vs. DMM 12w CCR2 +/+ | -69233 | -111479 to -26986 | **0.0006** |
| | DMM 8w CCR2 +/+ vs. DMM 12w CCR2 +/+ | -67764 | -110010 to -25518 | **0.0008** |
| ***% BV/TV*** | DMM 2w CCR2 +/+ vs. DMM 4w CCR2 +/+ | -3.402 | -8.930 to 2.126 | 0.3526 |
| | DMM 2w CCR2 +/+ vs. DMM 8w CCR2 +/+ | -7.509 | -13.04 to -1.981 | **0.0048** |
| | DMM 2w CCR2 +/+ vs. DMM 12w CCR2 +/+ | -12.3 | -17.83 to -6.770 | **<0.0001** |
| | DMM 4w CCR2 +/+ vs. DMM 8w CCR2 +/+ | -4.107 | -9.635 to 1.421 | 0.2019 |
| | DMM 4w CCR2 +/+ vs. DMM 12w CCR2 +/+ | -8.896 | -14.42 to -3.368 | **0.0008** |
| | DMM 8w CCR2 +/+ vs. DMM 12w CCR2 +/+ | -4.789 | -10.32 to 0.7389 | 0.1075 |

| OA Parameter Early Recomb. | Groups | Mean Diff. | 95.00% CI of diff. | Adjusted *P*-value |
|---|---|---|---|---|
| ***ACS*** | DMM 2w CCr2-Col1KO vs. DMM 4w CCr2-Col1KO | -4.531 | -7.259 to -1.804 | **0.0005** |
| | DMM 2w CCr2-Col1KO vs. DMM 8w CCr2-Col1KO | -6.313 | -9.040 to -3.585 | **<0.0001** |
| | DMM 2w CCr2-Col1KO vs. DMM 12w CCr2-Col1KO | -5.969 | -8.696 to -3.241 | **<0.0001** |
| | DMM 4w CCr2-Col1KO vs. DMM 8w CCr2-Col1KO | -1.781 | -4.509 to 0.9462 | 0.3023 |
| | DMM 4w CCr2-Col1KO vs. DMM 12w CCr2-Col1KO | -1.438 | -4.165 to 1.290 | 0.4866 |
| | DMM 8w CCr2-Col1KO vs. DMM 12w CCr2-Col1KO | 0.3438 | -2.384 to 3.071 | 0.9857 |
| ***Saf-O*** | DMM 2w CCr2-Col1KO vs. DMM 4w CCr2-Col1KO | -3.688 | -5.725 to -1.650 | **0.0002** |
| | DMM 2w CCr2-Col1KO vs. DMM 8w CCr2-Col1KO | -5.359 | -7.397 to -3.322 | **<0.0001** |
| | DMM 2w CCr2-Col1KO vs. DMM 12w CCr2-Col1KO | -4.969 | -7.006 to -2.931 | **<0.0001** |
| | DMM 4w CCr2-Col1KO vs. DMM 8w CCr2-Col1KO | -1.672 | -3.709 to 0.3655 | 0.1371 |
| | DMM 4w CCr2-Col1KO vs. DMM 12w CCr2-Col1KO | -1.281 | -3.319 to 0.7561 | 0.3341 |
| | DMM 8w CCr2-Col1KO vs. DMM 12w CCr2-Col1KO | 0.3906 | -1.647 to 2.428 | 0.9527 |
| ***Cartilage Histomorph.*** | DMM 2w CCr2-Col1KO vs. DMM 4w CCr2-Col1KO | 9081 | -2274 to 20436 | 0.1526 |
| | DMM 2w CCr2-Col1KO vs. DMM 8w CCr2-Col1KO | 12709 | 1353 to 24064 | **0.0238** |
| | DMM 2w CCr2-Col1KO vs. DMM 12w CCr2-Col1KO | 8136 | -3220 to 19491 | 0.2286 |
| | DMM 4w CCr2-Col1KO vs. DMM 8w CCr2-Col1KO | 3628 | -7727 to 14983 | 0.819 |
| | DMM 4w CCr2-Col1KO vs. DMM 12w CCr2-Col1KO | -945.3 | -12300 to 10410 | 0.9958 |
| | DMM 8w CCr2-Col1KO vs. DMM 12w CCr2-Col1KO | -4573 | -15928 to 6782 | 0.6927 |
| ***Subchondral Bone Thickness*** | DMM 2w CCr2-Col1KO vs. DMM 4w CCr2-Col1KO | -19020 | -64918 to 26877 | 0.6736 |
| | DMM 2w CCr2-Col1KO vs. DMM 8w CCr2-Col1KO | -34376 | -80273 to 11522 | 0.1961 |
| | DMM 2w CCr2-Col1KO vs. DMM 12w CCr2-Col1KO | 1646 | -44252 to 47543 | 0.9997 |
| | DMM 4w CCr2-Col1KO vs. DMM 8w CCr2-Col1KO | -15356 | -61253 to 30542 | 0.7979 |
| | DMM 4w CCr2-Col1KO vs. DMM 12w CCr2-Col1KO | 20666 | -25232 to 66563 | 0.6139 |
| | DMM 8w CCr2-Col1KO vs. DMM 12w CCr2-Col1KO | 36021 | -9876 to 81919 | 0.1645 |
| ***% BV/TV*** | DMM 2w CCr2-Col1KO vs. DMM 4w CCr2-Col1KO | -5.039 | -12.41 to 2.331 | 0.2649 |
| | DMM 2w CCr2-Col1KO vs. DMM 8w CCr2-Col1KO | -7.175 | -14.55 to 0.1953 | 0.0585 |
| | DMM 2w CCr2-Col1KO vs. DMM 12w CCr2-Col1KO | -4.313 | -11.68 to 3.057 | 0.3961 |
| | DMM 4w CCr2-Col1KO vs. DMM 8w CCr2-Col1KO | -2.136 | -9.506 to 5.235 | 0.8578 |
| | DMM 4w CCr2-Col1KO vs. DMM 12w CCr2-Col1KO | 0.7263 | -6.644 to 8.097 | 0.993 |
| | DMM 8w CCr2-Col1KO vs. DMM 12w CCr2-Col1KO | 2.862 | -4.508 to 10.23 | 0.716 |

**Supplemental Table S1. PTOA assessment in mouse knee joints of *CCR2-Col1αKO* and *CCR2+/+* within each genotype, following early Ccr2 inactivation.**

Within each genotype (***CCR2+/+* or *CCR2-Col1αKO*,** group analyses were performed with ordinary one-way ANOVA, followed by Tukey's post-hoc test for multiple comparisons.

**Supplemental Table S2. Effect of early CCR2 inactivation (before DMM) on synovial hyperplasia in mouse CCR2-Col1αKO and CCR2+/+ knee joints (N=8).**

| OA Parameters | | Week 2 | | | | Week 4 | | | |
|---|---|---|---|---|---|---|---|---|---|
| | | Z (p-value) | Mean Score (Rank Sums) | median | mean (SD) | Z (p-value) | Mean Score (Rank Sums) | median | mean (SD) |
| *Synovial Hyperplasia* | *DMM CCR2+/+* | 1.63(0.40) | 10.31 | 1 | 1.25(0.38) | -0.77 [0.88] | 7.56 | 1.25 | 1.31(0.37) |
| | *DMM CCR2-AggKO* | | 6.69 | 1 | 0.75(0.71) | | 9.44 | 1.75 | 1.50(0.60) |

| OA Parameters | | Week 8 | | | | Week 12 | | | |
|---|---|---|---|---|---|---|---|---|---|
| | | Z (p-value) | Mean Score (Rank Sums) | median | mean (SD) | Z (p-value) | Mean Score (Rank Sums) | median | mean (SD) |
| *Synovial Hyperplasia* | *DMM CCR2+/+* | 0.06(0.96) | 8.63 | 2 | 2.06(0.50) | 0.27(0.96) | 8.88 | 2.25 | 2.31(0.70) |
| | *DMM CCR2-AggKO* | | 8.38 | 2 | 2.00(0.60) | | 8.13 | 2.25 | 2.25(0.60) |

Data presented show median, mean and standard deviation (SD), for each group at separate time points (2, 4, 8 and 12 weeks post-surgery). Indicated *z*- and *P*-values were determined by Wilcoxon rank sum tests between DMM *CCR2+/+* and DMM *CCR2-Col1αKO* groups separately at each time point, following adjustment for multiple comparison (Benjamini-Hochberg).

**Supplemental Table S3.** **PTOA assessment in mouse knee joints of *CCR2-Col1αKO* and *CCR2*+/+ within each genotype, following late Ccr2 inactivation.**

| OA Parameter Late Recomb. | Groups | Mean Diff. | 95.00% CI of diff. | *P*-value |
|---|---|---|---|---|
| ***ACS*** | DMM 8w CCR2 +/+ vs. DMM 12w CCR2 +/+ | -3.09 | -5.45, -0.74 | **0.001** |
| ***Saf-O*** | DMM 8w CCR2 +/+ vs. DMM 12w CCR2 +/+ | -2.19 | -5.04, 0.664 | 0.12 |
| ***Cartilage Histomorph.*** | DMM 8w CCR2 +/+ vs. DMM 12w CCR2 +/+ | 3833 | -4852, 12518 | 0.34 |
| ***Subchondral Bone Thickness*** | DMM 8w CCR2 +/+ vs. DMM 12w CCR2 +/+ | -7521 | -54862, 39821 | 0.74 |
| ***% BV/TV*** | DMM 8w CCR2 +/+ vs. DMM 12w CCR2 +/+ | -4.41 | -9.41, 0.60 | 0.08 |

| OA Parameter Late Recomb. | Groups | Mean Diff. | 95.00% CI of diff. | *P*-value |
|---|---|---|---|---|
| ***ACS*** | DMM 8w CCr2-Col1KO vs. DMM 12w CCr2-Col1KO | 0.94 | -1.27, 3.15 | 0.37 |
| ***Saf-O*** | DMM 8w CCr2-Col1KO vs. DMM 12w CCr2-Col1KO | 1.08 | -0.46, 2.62 | 0.14 |
| ***Cartilage Histomorph.*** | DMM 8w CCr2-Col1KO vs. DMM 12w CCr2-Col1KO | -1554 | -10110, 7002 | 0.7 |
| ***Subchondral Bone Thickness*** | DMM 8w CCr2-Col1KO vs. DMM 12w CCr2-Col1KO | 21555 | -19894, 63005 | 0.28 |
| ***% BV/TV*** | DMM 8w CCr2-Col1KO vs. DMM 12w CCr2-Col1KO | -3.008 | -6.87, 0.85 | 0.12 |

Within each genotype (***CCR2*+/+ or *CCR2-Col1αKO)*** group analyses were performed with the original data with Welch's t-test.

**Supplemental Table S4. Effect of late CCR2 inactivation (4 weeks post-DMM) on synovial hyperplasia in mouse CCR2-Col1$\alpha$KO and CCR2+/+ knee joint (N=8).**

| OA Parameters | | Week 8 | | | | Week 12 | | | |
|---|---|---|---|---|---|---|---|---|---|
| | | Z (p-value) | Mean Score (Rank Sums) | median | mean (SD) | Z (p-value) | Mean Score (Rank Sums) | median | mean (SD) |
| *Synovial Hyperplasia* | *CCR2+/+* | -0.06 [0.96] | 8.38 | 2 | 1.69(0.75) | 0.32 [0.96] | 8.94 | 2.25 | 2.13(0.74) |
| | *CCR2-AggKO* | | 8.63 | 1.75 | 1.88(0.79) | | 8.06 | 2 | 2.0(0.58) |

Data presented show median, mean and standard deviation (SD), for each group at separate time points (8 and 12 weeks post-surgery). Indicated *z*- and *P*-values were determined by Wilcoxon rank sum tests between DMM *CCR2*+/+ and DMM *CCR2-Col1αKO* groups separately at each time point, following adjustment for multiple comparison (Benjamini-Hochberg).

**Supplemental Table S5. LMM results for incapacitance meter scores.** Estimated difference between groups, Standard Error, Adjusted P-values and lower and upper 95% confidence limits (LCL and UCL). Significant *P*-values ($\leq 0.05$) are indicated in bold.

| Time point | Group 1 | Group 2 | Estimate | Standard Error | Adj P | Adj Lower | Adj Upper |
|---|---|---|---|---|---|---|---|
| 4 weeks | DMM CCR2+/+ | DMM CCR2-Col1KO | 0.008247 | 0.02542 | 0.748 | -0.04381 | 0.06031 |
| | SHAM CCR2 +/+ | SHAM CCR2-Col1 KO | 0.02815 | 0.03077 | 0.3682 | -0.03489 | 0.09118 |
| | DMM CCR2+/+ | SHAM CCR2+/+ | -0.03526 | 0.02788 | 0.2163 | -0.09236 | 0.02184 |
| | DMM CCR2-Col1KO | SHAM CCR2-Col1KO | -0.01536 | 0.02856 | 0.5949 | -0.07387 | 0.04315 |
| 8 weeks | DMM CCR2+/+ | DMM CCR2-Col1KO | 0.143 | 0.05671 | **0.0177** | 0.02683 | 0.2592 |
| | SHAM CCR2 +/+ | SHAM CCR2-Col1 KO | 0.04836 | 0.06867 | 0.4871 | -0.0923 | 0.189 |
| | DMM CCR2+/+ | SHAM CCR2+/+ | 0.07648 | 0.0622 | 0.2291 | -0.05093 | 0.2039 |
| | DMM CCR2-Col1KO | SHAM CCR2-Col1KO | -0.01815 | 0.06374 | 0.778 | -0.1487 | 0.1124 |
| 12 weeks | DMM CCR2+/+ | DMM CCR2-Col1KO | 0.06495 | 0.03787 | 0.0974 | -0.01262 | 0.1425 |
| | SHAM CCR2 +/+ | SHAM CCR2-Col1 KO | 0.02656 | 0.04617 | 0.5697 | -0.06802 | 0.1211 |
| | DMM CCR2+/+ | SHAM CCR2+/+ | 0.06594 | 0.04205 | 0.1281 | -0.02019 | 0.1521 |
| | DMM CCR2-Col1KO | SHAM CCR2-Col1KO | 0.02755 | 0.04241 | 0.5213 | -0.05932 | 0.1144 |
| 16 weeks | DMM CCR2+/+ | DMM CCR2-Col1KO | 0.1472 | 0.05303 | **0.0097** | 0.03852 | 0.2558 |
| | SHAM CCR2 +/+ | SHAM CCR2-Col1 KO | -0.02212 | 0.06526 | 0.7371 | -0.1558 | 0.1116 |
| | DMM CCR2+/+ | SHAM CCR2+/+ | 0.1305 | 0.05681 | **0.0293** | 0.01414 | 0.2469 |
| | DMM CCR2-Col1KO | SHAM CCR2-Col1KO | -0.03878 | 0.062 | 0.5367 | -0.1658 | 0.08823 |
| 20 weeks | DMM CCR2+/+ | DMM CCR2-Col1KO | 0.1576 | 0.04078 | **0.0006** | 0.07406 | 0.2411 |
| | SHAM CCR2 +/+ | SHAM CCR2-Col1 KO | 0.01435 | 0.0483 | 0.7686 | -0.08459 | 0.1133 |
| | DMM CCR2+/+ | SHAM CCR2+/+ | 0.1348 | 0.04375 | **0.0046** | 0.04523 | 0.2245 |
| | DMM CCR2-AggKO | SHAM CCR2-Col1KO | -0.0084 | 0.04563 | 0.8552 | -0.1019 | 0.08506 |

**Supplemental Table S6. Between-group mean differences in Von Frey Analyses and 95% CIs.** Results are least-squares mean differences [ESTMATED MEAN in GROUP1 – ESTIMATED MEAN in GROUP2] and corresponding 95% confidence intervals from linear mixed effects model. Significant *P*-values (≤ *0.05*) are indicated in bold.

| Time point | Group1 | Group2 | DIFF_CI_BT | *P*-value |
|---|---|---|---|---|
| 4 weeks | Contral. CCR2 +/+ | DMM CCR2+/+ | -0.04 (-0.64,0.56) | 0.913167017 |
| | Contral. CCR2 +/+ | Contral. CCR2-Col1KO | -0.14 (-0.78,0.5) | 0.683122675 |
| | Contral. CCR2 +/+ | DMM CCR2-Col1KO | -0.37 (-1.07,0.33) | 0.306611043 |
| | DMM CCR2+/+ | Contral. CCR2-Col1KO | -0.1 (-0.74,0.54) | 0.765285234 |
| | DMM CCR2+/+ | DMM CCR2-Col1KO | -0.33 (-1.03,0.37) | 0.361282828 |
| | Contral. CCR2-Col1KO | DMM CCR2-Col1KO | -0.23 (-0.91,0.45) | 0.519439593 |
| 8 weeks | Contral. CCR2 +/+ | DMM CCR2+/+ | -0.02 (-0.64,0.59) | 0.945546013 |
| | Contral. CCR2 +/+ | Contral. CCR2-Col1KO | 0 (-0.62,0.62) | 0.998408783 |
| | Contral. CCR2 +/+ | DMM CCR2-Col1KO | -0.21 (-0.9,0.48) | 0.564371959 |
| | DMM CCR2+/+ | Contral. CCR2-Col1KO | 0.02 (-0.6,0.65) | 0.947672025 |
| | DMM CCR2+/+ | DMM CCR2-Col1KO | -0.19 (-0.88,0.51) | 0.610903109 |
| | Contral. CCR2-Col1KO | DMM CCR2-Col1KO | -0.21 (-0.88,0.46) | 0.552282224 |
| 12 weeks | Contral. CCR2 +/+ | DMM CCR2+/+ | 0.66 (0.19,1.14) | **0.006357264** |
| | Contral. CCR2 +/+ | Contral. CCR2-Col1KO | 0.2 (-0.34,0.73) | 0.47920658 |
| | Contral. CCR2 +/+ | DMM CCR2-Col1KO | 0.2 (-0.33,0.73) | 0.472760071 |
| | DMM CCR2+/+ | Contral. CCR2-Col1KO | -0.47 (-0.96,0.02) | 0.061238969 |
| | DMM CCR2+/+ | DMM CCR2-Col1KO | -0.46 (-0.95,0.03) | 0.062683876 |
| | Contral. CCR2-Col1KO | DMM CCR2-Col1KO | 0 (-0.45,0.45) | 0.991157927 |
| 16 weeks | Contral. CCR2 +/+ | DMM CCR2+/+ | 0.44 (0.02,0.86) | **0.037585934** |
| | Contral. CCR2 +/+ | Contral. CCR2-Col1KO | -0.07 (-0.52,0.39) | 0.790616054 |
| | Contral. CCR2 +/+ | DMM CCR2-Col1KO | 0.42 (-0.03,0.87) | 0.064456736 |
| | DMM CCR2+/+ | Contral. CCR2-Col1KO | -0.51 (-0.96,-0.05) | 0.027903491 |
| | DMM CCR2+/+ | DMM CCR2-Col1KO | -0.02 (-0.4,0.36) | 0.930101194 |
| | Contral. CCR2-Col1KO | DMM CCR2-Col1KO | 0.49 (0.07,0.91) | **0.023037616** |
| 20 weeks | Contral. CCR2 +/+ | DMM CCR2+/+ | 0.29 (-0.08,0.65) | 0.12453398 |
| | Contral. CCR2 +/+ | Contral. CCR2-Col1KO | 0.13 (-0.27,0.53) | 0.537568916 |
| | Contral. CCR2 +/+ | DMM CCR2-Col1KO | 0.13 (-0.27,0.53) | 0.547252234 |
| | DMM CCR2+/+ | Contral. CCR2-Col1KO | -0.16 (-0.54,0.22) | 0.424023015 |
| | DMM CCR2+/+ | DMM CCR2-Col1KO | -0.16 (-0.54,0.22) | 0.41560958 |
| | Contral. CCR2-Col1KO | DMM CCR2-Col1KO | 0 (-0.33,0.33) | 0.987231178 |

**Supplemental Table S7. Comparison between Early vs Late Recombination on PTOA patameters at 8 and 12 weeks post DMM in mouse CCR2-Col1αKO and CCR2+/+ knee joint (N=8).**

| OA Parameters | | Week 8 | | | | Week 12 | | | |
|---|---|---|---|---|---|---|---|---|---|
| | | Z (p-value) | Mean Score (Rank Sums) | median | mean (SD) | Z (p-value) | Mean Score (Rank Sums) | median | mean (SD) |
| *ACS* | Early recombination | 0.74 (0.46) | 9.44 | 7.5 | 7.13(1.76) | 1.05 (0.46) | 9.81 | 7.25 | 6.78(3.29) |
| | Late Recombination | | 7.56 | 6.38 | 6.47(1.28) | | 7.19 | 5.5 | 5.53(2.51) |
| *Saf-O* | Early recombination | -1.27 (0.41) | 6.94 | 6.63 | 6.42(1.62) | 0.29 (0.77) | 7.63 | 6.13 | 6.03(2.18) |
| | Late Recombination | | 10.06 | 7.5 | 7.44(0.75) | | 8.43 | 7 | 6.36(1.63) |
| *Cartilage Histomorphometry* | Early recombination | -1.1 (0.54) | 7.13 | 18217 | 18364(9114) | -0.37 (0.71) | 8 | 20436 | 22937(10655) |
| | Late Recombination | | 9.88 | 26515 | 24075(8390) | | 9 | 24930 | 25629(7527) |
| | | | | | | | | | |
| *Osteophyte Cartilage Quantification* | Early recombination | 0 (1.00) | 8.5 | 34.9 | 39.61(13.39) | -1.94 (0.10) | 6.13 | 38.17 | 35.77(11.86) |
| | Late Recombination | | 8.5 | 38.43 | 38.27(10.71) | | 10.88 | 45.78 | 46.90(7.85) |
| *Osteophyte Size* | Early recombination | 0.83 (0.41) | 9.5 | 1.25 | 1.22(0.25) | 0.92 (0.41) | 9.63 | 1.25 | 1.22(0.36) |
| | Late Recombination | | 7.5 | 1.13 | 1.13(0.33) | | 7.38 | 1 | 1.06(0.35) |
| *Osteophyte Maturity* | Early recombination | 0.5 (0.62) | 9.13 | 1 | 1.06(0.32) | 1.57 (0.23) | 10.31 | 1.13 | 1.28(0.43) |
| | Late Recombination | | 7.88 | 1 | 0.94(0.26) | | 6.69 | 1 | 0.94(0.22) |
| | | | | | | | | | |
| *%BV over TV* | Early recombination | 0.89 (0.37) | 9.63 | 88.39 | 87.67(5.25) | -1.31 (0.37) | 6.88 | 84.99 | 84.81(5.55) |
| | Late Recombination | | 7.38 | 85.61 | 85.39(4.08) | | 10.13 | 88.37 | 88.40(2.96) |
| *Subchondral Bone Thickness* | Early recombination | -0.68 (0.50) | 7.63 | 159878 | 154086(28876) | -1.94 (0.14) | 6.13 | 113190 | 118065(19106) |
| | Late Recombination | | 9.38 | 168541 | 173802(37031) | | 10.88 | 161004 | 152247(40161) |
| | | | | | | | | | |
| *Synovial Hyperplasia* | Early recombination | 0.49 (0.62) | 9.13 | 2 | 2.00(0.60) | 0.71 (0.62) | 9.38 | 2.25 | 2.25(0.60) |
| | Late Recombination | | 7.88 | 1.75 | 1.88(0.79) | | 7.63 | 2 | 2.00(0.60) |

Data presented show median, mean and standard deviation (SD), for each group at separate time points (8 and 12 weeks post-surgery). Indicated *z*- and *P*-values were determined by Wilcoxon rank sum tests between DMM *CCR2+/+* and DMM *CCR2-Col1αKO* groups separately at each time point, following adjustment for multiple comparison (Benjamini-Hochberg).
